# An Evaluation of Alternatives to Nitrites and Sulfites to Inhibit the Growth of *Salmonella enterica* and *Listeria monocytogenes* in Meat Products

**DOI:** 10.3390/foods5040074

**Published:** 2016-10-31

**Authors:** Alexandre Lamas, José Manuel Miranda, Beatriz Vázquez, Alberto Cepeda, Carlos Manuel Franco

**Affiliations:** Laboratorio de Higiene Inspección y Control de Alimentos, Dpto. de Química Analítica, Nutrición y Bromatología, Universidad de Santiago de Compostela, 27002-Lugo, Spain; alexandre.lamas@usc.es (A.L.); josemanuel.miranda@usc.es (J.M.M.); beatriz.vazquez@usc.es (B.V.); alberto.cepeda@usc.es (A.C.)

**Keywords:** *Salmonella*, *Listeria*, nitrites, sulfites, sodium acetate, chitosan, meat

## Abstract

In recent years, the use of nitrites and sulfites as food preservatives has been a cause for concern due to the health problems that these additives can cause in humans. Natural products have been studied as an alternative, but most of them have hardly been applied in the food industry for technological and economic reasons. In this sense, organic salts such as sodium acetate are a good alternative due to their affordability. Thus, this study evaluated the capacity of sodium nitrite, sodium sulfite, a sodium acetate product (TQI C-6000), and chitosan to inhibit two important foodborne pathogens, *Salmonella enterica* and *Listeria monocytogenes*. The MIC of each chemical was in vitro evaluated and their antibacterial action was subsequently checked in situ using minced meat as a food model. MIC values of sodium nitrite (10,000 mg/L) and sodium sulfite (50,000 mg/L) for *Salmonella enterica* were higher than the values allowed by legislation (450 mg/L for sulfites and 150 mg/L for nitrites). Additionally, the sodium acetate product caused the inhibition of *Salmonella enterica* and *Listeria* at a relative low quantity. The two foodborne pathogens were inhibited in the food model with 1% of the sodium acetate product. Additionally, there were no significant differences between sodium nitrite, sodium sulfite, and sodium acetate products in the inhibition of *Salmonella enterica* and *Listeria monocytogenes* in the food model. Thus, products based on sodium acetate can be an alternative to traditional preservatives in food products.

## 1. Introduction

Some of the main concerns about meat products are the microbial spoilage and oxidation because they are responsible for a loss in quality and the development of toxic compounds that can affect human health [1]. In the same way, the growth of foodborne pathogens, since the meat is stored until it is purchased by the consumer, is a public health problem [2]. Commission Regulation (EU) 1129/2011 establishes a positive list of additives that can be used in food products [3]. There are different types of additives with different functions such as the preservation of food by protecting against deterioration caused by microorganisms. In this sense, nitrites and sulfites are two of the most common preservatives used in meat products that prevent the growth of microorganisms [4,5].

Sulfites have some antimicrobial and antioxidant properties and act as color stabilizers [4]. Nitrites have some advantages, such as contributing to the preservation of the meat. They are responsible for the development of a typical curing color and flavor, and they inhibit the growth of pathogenic bacteria [5]. However, the use of these preservatives has some drawbacks that have to be considered. Sulfites could be responsible for allergic and respiratory reactions in sensitive people and chronic skin symptoms [6]. Nitrites are toxic at high concentrations and can form nitrosamines when exposed to certain heating or acid conditions [7]. Taking this under consideration, the amount of nitrites and sulfites added to meat products are legally restricted in the European Union. Thus, nitrites and sulfites are in the spotlight more than ever because in recent years consumers have been seeking products that do not cause health problems. In this sense, some studies have evaluated the reduction of the amount of nitrites and sulfites necessary to avoid the growth of foodborne pathogens [4,8]. In this way, there has been much research focused on the evaluation of natural alternatives that can substitute traditional preservatives [9,10,11]. However, these natural additives are hard to apply in the food industry for technological and economic reasons. Hence, some authors have evaluated alternative additives such as organic acid salts as substitutes of traditional preservatives [12]. 

*Salmonella enterica* subspecies *enterica* and *Listeria monocytogenes* are ones of the main microorganisms responsible for foodborne diseases around the world. In 2014, in the European Union (EU), there were 88,715 confirmed cases of salmonellosis and 2161 of listeriosis [13]. Additionally, it is worrying that the incidence of both has increased in the last several years in the EU [13]. *S. enterica* and *L. monocytogenes* are isolated, among other foods, from meat products such as broilers, pork, and beef meat [14,15,16]. Thus, in some cases, the use of substances that help to inhibit the growth of *Salmonella enterica* and *L. monocytogenes* in these foods is important.

Amounts of nitrites and sulfites that can be added to food are legislated. Previous studies were focused only in alternatives to these preservatives. However, it is also important to determine whether a certain amounts of nitrites and sulfites are in fact effective against foodborne pathogens, given the adverse effects they can produce in humans. Thus, an important aim of this study was to evaluate, both in vitro and food model studies, the real capacity of these two traditional preservatives to inhibit the growth of foodborne pathogens. At the same time, another aim was to determine whether alternative preservatives based in the use of sodium acetate and without adverse effects could be a feasible alternative of these traditional preservatives. Thus, the study was divided in two parts. First, we evaluated in vitro the minimum inhibitory concentrations of chemicals against *S. enterica* and *L. monocytogenes*. Then, we evaluated the same additives in a food model at the maximum level allowed by legislation in the case of nitrites and sulfites and at a level that shows inhibition in vitro tests for sodium acetate and chitosan. The variation of pH after the addition of chemicals was also evaluated during the storage.

## 2. Materials and Methods

### 2.1. Bacterial Strains and Additives

*Salmonella* Typhimurium CECT 4594 and 3 strains isolated from poultry houses [17] corresponding to *Salmonella* Enteritidis, *Salmonella* Infantis, and *Salmonella* Newport were used for inhibition studies. *L. monocytogenes* CECT 934 and three *L. monocytogenes* strains isolated from meat, coded as Lhica 1, Lhica 4, and Lhica 7, were selected for the study (Table 1). Sodium nitrite (Sigma-Aldrich, Darmstadt, Germany) and sodium sulfite (Sigma-Aldrich) were selected as representatives of nitrites and sulfites. A food grade product composed of sodium acetate and citric acid (TQI C-6000, TEQUISA, Pontevedra, Spain) and food grade chitosan (ChitoClear^®^, Primex, Siglufjordur, Iceland) were used for this study.

### 2.2. Minimum Inhibitory Concentration of Additives Against S. enterica and L. monocytogenes

Minimal inhibitory concentration (MIC) testing was performed by microdilution broth method according to the guidelines of Clinical and Laboratory Standards Institute [18]. Briefly, *S. enterica* and *L. monocitogenes* strains were cultured in Brain Heart Infusion Agar plates (BHI, Oxoid, Hampshire, UK) and incubated for 24 h at 37 °C. Then, bacterial suspensions were prepared in saline solution at McFarland 0.5 density (10^8^ cfu/mL) and diluted to 10^6^ cfu/mL in a saline solution. The Mueller–Hilton broth was used to prepare two-fold dilution series of each preservative tested. The wells of a microplate were filled with the preservative solutions and 10 µL of the bacterial inoculum at a final concentration of 10^5^ cfu/mL of tested microorganism in each well. Microplates were incubated at 37 °C for 24 h. After incubation, the lowest concentration of the additives that completely inhibited the macroscopic growth was determined as MIC. Each strain was tested in triplicate in three different experiments.

### 2.3. Preparation and Inoculation of Minced Meat

Inoculation of minced meat with *S. enterica* and *L. monocytogenes* was made separately for each foodborne pathogen. The minced meat was purchased from the local market and was composed of 50% pork meat and 50% beef meat. For each batch, five individual stomacher bags of 25 g were elaborated. Then, each additive was added to each batch of meat and homogenized. Five different batches of minced meat were elaborated: (1) control meat (CM); (2) sulfite meat (SM); (3) nitrite meat (NM); (4) sodium acetate meat (AM); (5) sodium acetate–chitosan meat (CHM). For sulfite and nitrite, the concentration added was the maximum allowed by legislation, 450 mg/kg for sulfites and 150 mg/kg for nitrites. In the case of the sodium acetate product and the sodium acetate product–chitosan, the concentrations added were calculated according to the results of MIC tests. Thus, 1% AM and 1.25% CHM (1% sodium acetate and 0.025% chitosan) was added to each batch of minced meat.

The results obtained in the MIC tests showed little differences between the strains of each group of pathogens. Thus, the *Salmonella* CECT 4594 and *L. monocytogenes* Lhica 4 strains were selected as representative strains to inoculate the meat. Briefly, these two strains were cultured in BHI plates and incubated for 24 h at 37 °C. Then, bacterial suspensions at McFarland 0.5 density (10^8^ cfu/mL) were prepared and diluted. For the *S. enterica* and *L. monocytogenes* inhibition study, the stomacher bag was inoculated with 1 mL of the final dilution of the adequate pathogen in each case to a final concentration of 3 log cfu/g in the minced meat. Finally, the meat batches were conserved at a cooling temperature (4–6 °C) during the entire study. Assays were carried out in triplicate.

### 2.4. Microbiological Analysis

Microbiological analysis was performed each three days. The absence of *Salmonella* spp. and *L. monocytogenes* in the meat samples was previously verified. To ensure the absence of the *Salmonella* spp. in meat, 25 g of minced meat were homogenized with 225 mL of peptone water and incubated for 18 h at 37 °C. Then, 0.1 mL of incubated peptone water were transferred to a tube with 10 mL of Rappaport-Vassiliadis Soya Peptona broth (Merck, Darmstadt, Germany) and incubated at 42 °C for 24 h, and this media was finally harvested using an inoculating loop in XLD (Oxoid) and SM-ID2 (bioMérieux, Marcy-l'Étoile, France) to detect the presence of presumptive *Salmonella* spp. colonies. To ensure the absence of *Listeria monocytogenes* in meat, 25 g of minced meat were incubated in half Fraser broth (Oxoid) at 30 °C for 24 h. Then, 0.1 mL were transferred to a tube containing 10 mL of Fraser broth and incubated at 37 °C for 48 h. Finally, the half- and full-strength Fraser broths were plated out on Aloa agar (bioMérieux) and incubated at 37 °C for 48 h to detect the presence of presumptive *L. monocytogenes*. For the other microbiologic analysis, 25 g of minced meat were homogenized with 225 mL of buffered peptone water (Merck) for two minutes in a stomacher (MIX2, AES-Laboratory, Combourg, France). Total viable counts were evaluated on a Plate Count Agar (Liofilchem, Roseto degli Abruzzi, Italy) and incubated at 32 °C for 72 h. Entobacteriaceae were counted in Violet Red Bile Glucose Agar (Liofilchem) and incubated at 37 °C for 24 h. *Escherichia coli* were determined in Fluorocult^®^ (Merck) and incubated at 42 °C for 24 h. The presence of presumptive *Staphylococcus aureus* was evaluated in Baird Parker agar (bioMérieux) and incubated at 37 °C for 48 h. *Salmonella* were determined in XLD (Oxoid) at 37 °C for 24 h. *L. monocytogenes* were counted in Aloa agar (bioMérieux) and incubated at 37 °C for 48 h.

### 2.5. pH Measurement

Measurement of pH was carried out in a Crison PH 25+ pH meter with a penetration electrode (Crison Instruments, Barcelona, Spain) by introducing the electrode in the minced meat. Determinations for each treated minced meat were performed in triplicate every 3 days.

### 2.6. Statistical Analysis

Statistical analyses were performed using SPSS software for Windows (SPSS Inc., Chicago, IL, USA). An analysis of variance (ANOVA) was used to study the influence of the different additives added to the minced meat on the growth of microorganisms and the pH during storage.

## 3. Results

### 3.1. Minimum Inhibitory Concentration of Additives Against S. enterica and L. mnocytogenes

The minimum inhibitory concentration (MIC) of sodium nitrite, sodium sulfite, sodium acetate, and chitosan was determined against four *S. enterica* and four *L. monocytogenes* strains (Table 1). The inhibition of sodium nitrite was very different between the two microbiological species tested. All *S. enterica* strains showed a MIC of 10,000 mg/kg, a value far exceeding the 150 mg/kg allowed in food by legislation. Two of the *L. monocytogenes* strains tested in this study showed a MIC of 125 mg/kg, and the other two strains showed a MIC of 300 mg/kg. Therefore, only two of the four *Listeria monytogenes* strains tested in this study were inhibited at levels below those allowed by legislation for nitrites in meat products (150 mg/kg). For sodium sulfite, the inhibition was also very different between *S. enterica* and *L. monocytogenes*. However, in this case, all strains had a MIC higher than 450 mg/kg allowed by legislation. The four *S. enterica* strains had a MIC of 50,000 mg/kg and the four *L. monocytogenes* strains had a MIC of 625 mg/kg. It is remarkable that sodium acetate and citric acid (TQI C-6000) had a lower MIC (5000 mg/kg) for *S. enterica* strains than did sodium nitrite and sodium sulfite. However, for *L. monocytogenes* strains, the MIC value (1250 mg/kg) was higher than for sodium nitrite and sodium sulfite. Finally, food grade chitosan had a MIC value of 250 mg/kg against the four *S. enterica* strains and two *L. monocytogenes* strains, and a MIC of 125 mg/kg against the other two *L. monocytogenes* strains.

### 3.2. Microbiology of Minced Meat

**Figure 1:**
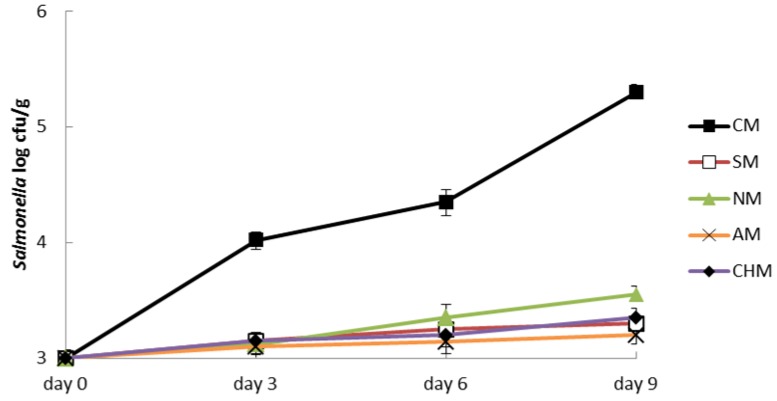
Changes in *S.* Typhimurium CECT 4594 inoculated in minced meat during storage at 4–6 °C. Control meat (CM), sodium meat (SM), nitrite meat (NM), sodium acetate meat (AM), sodium acetate–chitosan meat (CHM).

The minced meat was stored at a cooling temperature (4–6 °C) for 9 days. The microbiological analyses were performed on days 0, 3, 6, and 9. There were significant differences (*p* < 0.05) in the growth of *S. enterica* between the control batch and the other four different batches (Figure 1). In CM, *S. enterica* evolved from 3 cfu. log/g at the beginning to 5.30 log cfu/g at the end of the study. However, in the other four different batches with additives *S. enterica* growth was just 0.30 log cfu/g of minced meat. There were no significant differences (*p* > 0.05) between these four treatments, but the lower *S. enterica* growth was with sodium acetate with a final level of 3.20 log cfu/g.

**Figure 2:**
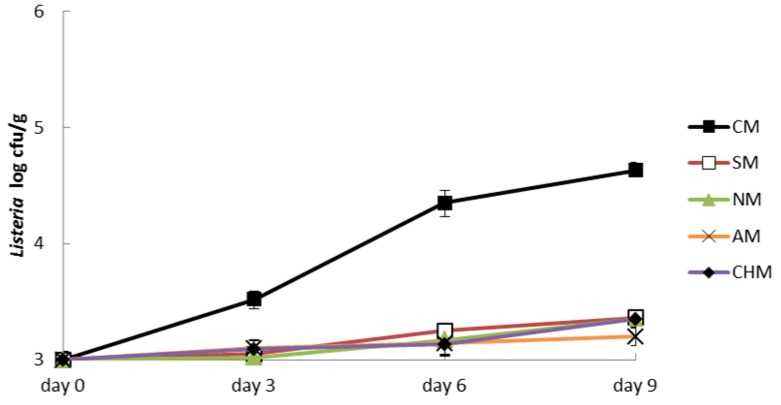
Changes in *L. monocytogenes* Lhica 4 inoculated in minced meat during storage at 4–6 °C. Control meat (CM), sodium meat (SM), nitrite meat (NM), sodium acetate meat (AM), sodium acetate–chitosan meat (CHM).

*L. monocytogenes* followed a behavior similar to *S. enterica* (Figure 2). Thus, there were significant differences (*p* < 0.05) between CM and the other four batches with additives. CM had an initial contamination of 3 log cfu/g and a final contamination of 4.63 log cfu/g. In this case, the final growth of *L. monocytogenes* was lower than *S. enterica* growth. There were no significant differences between the four batches treated with additives; however, as before, AM had lower *L. monocytogenes* growth with a final level of 3.21 log cfu/g.

The increase in other microorganisms initially present in the food in terms of total viable counts, Enterobacteriaceae, *E. coli*, and *S. aureus* was also evaluated in the minced meat contaminated with *S. enterica* (Table 2) and *L. monocytogenes* (Table 3). The levels of *S. aureus* in minced meat were lower than the detection limit of the applied method (100 cfu/g) during the entire study, and their growth could not be evaluated. The results showed a great difference between the different additives in the growth of the microorganisms. CHM and AM were the treatments more efficient showing lower counts in total viable counts, Enterobacteriaceae, and *E. coli* plates. It is interesting that there were no significant differences (*p* > 0.05) between CHM and AM treatments. The increase in total viable count was lower than 2 log cfu/g for CHM and AM in both *S. enterica* and *L. monocytogenes* batches and had a difference of 2 log cfu/g with CM after nine days. The differences of NM and SM were approximately 0.5 log cfu/g and 1 log cfu/g, respectively. In the case of Enterobacteriaceae, there was a great difference in the growth of that group of microorganism between AM and CHM and the others batches. Thus, Enterobacteriaceae did not grow in the AM and CHM batches, and there were no significant differences (*p* > 0.05) in initial and final counts. However, Enterobacteriaceae had a change of 2 log cfu/g in the NM batch and 2.5 log cfu/g in the SM batch. Although NM and SM caused less inhibition of Enterobacteriaceae than AM and CHM, the growth of that group of microorganism was lower in NM and SM than in CM. In the case of *E. coli*, there was an evolution of 0.5 log ufc/g in AM and CHM batches, 1 log ufc/g in NM, 1.5 log ufc/g in SM, and 2 log ufc/g in CM. Therefore, AM and CHM were the treatments with higher inhibition. Likewise, NM was more efficient than SM to control the growth of microorganisms.

### 3.3. pH Measurement

There were significant differences (*p* < 0.05) in minced meat pH after adding the different treatments (Table 4). After adding the sodium acetate and citric acid products, the pH decreased significantly in the minced meat; in CM, the pH was 5.64 ± 0.12 in AM—5.07 ± 0.06 at day 0. At day 9, there were significant differences between the different batches. CM had a higher pH (7.42 ± 0.08), and NM and SM had pH values of 7.05 ± 0.12 and 7.10 ± 0.05, respectively. AM and CHM had the lowest pH values at the end of the study—5.92 ± 0.06 and 5.96 ± 0.09, respectively.

## 4. Discussion

Meat and meat products are characterized by pH, water activity, and the level of nutrients that cause the easy growth of microorganisms during their storage [19]. Sulfites and nitrites are two common additives used in the food industry for meat preservation and to avoid the growth of foodborne pathogens [20,21]. However, in recent years, some studies have been focused on the substitution of these additives by acid salts or natural products without considering safety [1,4,20]. Thus, in this study, the effectiveness of a sodium acetate product alone and in combination with chitosan was compared with sodium nitrite and sodium sulfite to prevent the growth of *S. enterica* and *L. monocytogenes* strains both in vitro and in a food model.

In vitro results showed a higher resistance of *S. enterica* strains to additives compared with *L. monocytogenes* strains. *S. enterica* strains had a MIC for sodium nitrite (10,000 mg/kg) and sulfite (50,000 mg/kg) significantly higher than values allowed for these additives in meat [3]. In the same way, the results of this study differ from those of Buket et al. [22], who obtained a MIC of 250 mg/kg for a *S. enterica* strain, a value much lower than our results. The differences between these two studies could be due to the presence of determinate mechanisms in the *Salmonella* strains tested, allowing for a better tolerance to sodium nitrite. Therefore, the use of these two preservatives to prevent the growth of foodborne pathogens such as *S. enterica* is at best doubtful according to in vitro results. Sodium acetate had a MIC of 5000 mg/kg for *S. enterica* strains. This value is similar to the value obtained by Milillo and Ricke [12] in a study using BHI media and chicken juice. The fact that sodium acetate has a lower MIC value than sodium sulfite and nitrite makes it a good alternative for their use in food to prevent *S. enterica* growth. Food grade chitosan had a lower MIC value (250 mg/kg) in *S. enterica*. The antimicrobial and the antioxidant effects of chitosan make it a good alternative to traditional preservatives [4]. However, its high price is a limitation that inhibits its wide use in the food industry. A good solution could be the combination of low amounts of chitosan with other preservatives such as sodium acetate that has a more affordable price. Chitosan needs acid conditions to dissolve well in aqueous solution; for this reason, a 0.1% acetic acid solution is usually the better choice to dissolve it. TQI C-6000 due to its content of sodium acetate with citric acid creates a pH solution of 4.2, which allows the dissolution of chitosan, making it possible to avoid the use of acetic acid.

*L. monocytogenes* strains had very lower MIC values for all the preservatives in comparison with *S. enterica,* except in the case of the chitosan. Thus, two of the four strains tested had a MIC for sodium nitrite lower than the maximum amount allowed by legislation [3]. Therefore, the capacity of the traditional preservative sodium nitrite seems to be more useful for controlling the growth of *L. monocytogenes* in meat products. It is possible that *S. enterica* have some metabolic characteristics that make this microorganism more resistant to sodium nitrite and sodium sulfite. The MIC values of sodium acetate were 1250 mg/kg for the four *L. monocytogenes* strains. Neetoo et al. [23] had similar results in a study that tested some preservatives for the inhibition of the growth of *L. monocytogenes* in salmon products. Thus, sodium acetate also showed its potential to control the growth of *L. monocytogenes* with a MIC 25% of *S. enterica.* The chitosan MIC for *L. monocytogenes* was 125 and 250 mg/kg, similar to the values obtained for other microorganisms in other studies that tested chitosan with different molecular weights [24].

The MIC values obtained in vitro need to be evaluated in food models to corroborate the results. Food is a complex matrix with a microbiota that interacts between each other. These interactions together with the presence of other substances such as fat or protein may affect the efficacy of the preservatives to inhibit growth of pathogens. Minced meat was used as a food model in this study. Sodium nitrite and sodium sulfite were independently used at the maximum level allowed by legislation. The MIC results of sodium acetate showed inhibition of both pathogens at a concentration of 5000 mg/kg. Food is a complex matrix with different groups of microorganisms, and sodium acetate was added to comprise 1% of minced meat to ensure its effect. Then, the other batch was elaborated with sodium acetate combined with chitosan due to their antimicrobial and antioxidant activities.

The results of this study showed a great difference between the MIC values observed in a pure culture of the pathogens tested that were incubated at optimum temperatures and inhibition of these pathogens caused by these preservatives in a food model that was stored at cooling temperature. Therefore, MIC values cannot be directly extrapolated to a food model. Meanwhile, MIC values of sodium nitrite and sodium sulfite in *S. enterica* were very high compared to the levels added to the food (450 mg/kg of sodium sulfite and 150 mg/kg of sodium nitrite). However, with these levels, it was possible to control the growth of foodborne pathogens in a food model. Thus, there were no significant differences in the inhibition of *S. enterica* and *L. monocytogenes* growth with the four additives tested. Additionally, the high difference in the in vitro inhibition of *S. enterica* and *L. monocytogenes* caused by sodium nitrite and sodium sulfite was no observed in the food model. The difference between the in vitro analyses and food models is clear. Herbst et al. [5] observed that acidified nitrite caused an improved inhibition of *L. monocytogenes*. They observed that a pH below 6 affects the growth of this pathogen. In the NM batch, the pH on day 0 was 5.73 ± 0.04. Thus, this lower pH could be responsible for the difference found between in vitro analysis and food models. Additionally, Gutierrez et al. [25] tested the effectiveness of some essential oils in vitro and of food models. They observed, in some cases, improved inhibition of essentials oils in the food models. They explained that the presence of food components such as proteins and fat, or pH, could affect the effectiveness of these antimicrobials. Vogel et al. [26] found that 0.21% sodium acetate caused no inhibition in *L. monocytogenes* growth in cold-smoked salmon. However, in this study, we have inhibition in minced meat using 1% sodium acetate. Increasing the amount of sodium acetate to food and their combination with citric acid (TQI C-6000), which caused a decline in pH values, helps to control the growth of *L. monocytogenes*. Serdengecti et al. [27] found *Salmonella* Enteritidis inhibition in minced beef with 0.1% sodium acetate; however, this amount did not cause inhibition of *L. monocytogenes.* Thus, values of 1% sodium acetate could be a good alternative to traditional preservatives such as sodium nitrite and sulfite to inhibit the growth of foodborne pathogens in food during storage. In the same way, the combination of sodium acetate with chitosan also caused the inhibition of *S. enterica* and *L. monocytogenes* growth. Thus, chitosan could be used to maintain antioxidant activities that sulfites have in food.

However, in this study, significant differences were found between the four additive combinations with respect to total viable count, Enterobacteriaceae, and *E. coli*. AM and CHM caused more inhibition on the growth of these three groups of microorganisms. At the end of the study, AM and CHM had two logs less than the CM batch and one log less than the SM in the total viable count. AM and CHM also had the lowest pH values during 9 days of storage. Thus, it is possible that this lower pH could be responsible for the differences found in microbiological analyses. If we focus on Enterobacteriaceae, the capability of AM and CHM to inhibit their growth was greater, with almost two and three logs less than the traditional preservatives sodium nitrite and sulfite, respectively. It is possible that the acidification in food caused by sodium acetate and citric acid combination was responsible for the lower microbial growth in AM and CHM since day 0 of the study. Although sodium nitrite and sulfite showed a similar potential to inhibit the growth of *L. monocytogenes* and *S. enterica* in the food model, their capacity to inhibit the growth of other microbial groups was lower than TQI C-6000 preservative capacity. Chitosan has been studied in this work because of their antimicrobial capacity [4,24]. However, the results obtained in this study showed that adding chitosan with TQI C-6000 did not increase the antimicrobial activity of this product in food. However, their other functions such as antioxidant activity recommend their use in food [28]. Other studies carried out in fish and beef patties have also observed decreases in bacterial count when sodium acetate was used [29,30]. Therefore, the use of sodium acetate combined with citric acid (TQI C-6000) or with chitosan is a good choice that can improve the shelf life of meat products better than nitrites and sulfites. Additionally, sodium acetate, similar to the other two traditional preservatives, does not require any concern with respect to human health.

## 5. Conclusions

In recent years, the use of preservatives such as sulfites and nitrites has been largely discussed due to the health problems that they can cause. However, their use is still necessary due to their antimicrobial properties. The need for providing healthy and safe products to the consumer makes the task of looking for alternatives necessary. Although it is possible to find research testing natural products as alternatives to nitrites and sulfites, these natural products are normally difficult to obtain and not applicable at the industry level. Thus, this study showed that products composed principally of sodium acetate such as TQI C-6000 could be used as a safe and relatively cheap alternative to sulfites and nitrites. In vitro and food model studies showed the ability of this alternative to control the growth of *L. monocytogenes* and *S. enterica*. Likewise, sodium acetate, compared with sodium nitrite and sulfite, showed a better capacity to inhibit the growth of spoilage bacteria. Their combination with chitosan could replace the antioxidant activity of sulfite in food when it is added. Consequently, the food industry should evaluate the substitution of nitrites and sulfites by sodium acetate-based products.

## Figures and Tables

**Table 1:** Minimum inhibitory concentration (MIC) for each additive tested in *Listeria monocytogenes* and *Salmonella enterica* strains used in this study. Results are expressed as mg/L.

Strain	Sodium Nitrite	Sodium Sulfite	TQI C-6000	Chitosan
*S.* Typhimurium CECT 4594	10,000	50,000	5000	250
*S.* Enteritidis Lhica 1	10,000	50,000	5000	250
*S.* Infantis Lhica 2	10,000	50,000	5000	250
*S.* Newport Lhica 4	10,000	50,000	5000	250
*L. monocytogenes* CECT 934	125	625	1250	125
*L. monocytogenes* Lhica 1	125	625	1250	125
*L. monocytogenes* Lhica 4	300	625	1250	250
*L. monocytogenes* Lhica 7	300	625	1250	250

**Table 2:** Microbial changes in minced meat contaminated with *Salmonella* CECT 4594 in the five different batches elaborated during storage time. Results are expressed as log cfu/g. Different superscript letters in the same row indicate significant differences (*p* < 0.05). Different subscript letters in the same column and microbial group indicate significant differences (*p* < 0.05).

Microbial Group	Storage Day	Batch				
		CM	SM	NM	AM	CHM
Total viable count	0	5.47 ± 0.01 _a_	5.40 ± 0.02 _a_	5.55 ± 0.01 _a_	5.50 ± 0.01 _a_	5.45 ± 0.01 _a_
	3	7.46 ± 0.15 ^d^_b_	6.70 ± 0.13 ^c^_b_	6.17 ± 0.19 ^b^_b_	5.47 ± 0.16 ^a^_a_	5.58 ± 0.02 ^a^_b_
	6	7.95 ± 0.23 ^c^_c_	6.82 ± 0.14 ^b^_b_	6.78 ± 0.11 ^b^_c_	6.08 ± 0.18 ^a^_b_	6.18 ± 0.04 ^a^_c_
	9	8.83 ± 0.12 ^d^_d_	7.85 ± 0.11 ^c^_c_	7.39 ± 0.17 ^b^_d_	6.79 ± 0.11 ^a^_c_	6.84 ± 0.06 ^a^_d_
Enterobacteriaceae	0	3.88 ± 0.02 _a_	3.89 ± 0.02 _a_	3.87 ± 0.03 _a_	3.92 ± 0.02 _a_	3.90 ± 0.02 _a_
	3	4.83 ± 0.12 ^b^_b_	4.25 ± 0.14 ^a^_b_	4.15 ± 0.12 ^a^_b_	4.13 ± 0.11 ^a^_b_	4.18 ± 0.19 ^a^_b_
	6	6.51 ± 0.04 ^d^_c_	5.21 ± 0.09 ^c^_c_	4.71 ± 0.18 ^b^_c_	4.11 ± 0.07 ^a^_b_	4.15 ± 0.12 ^ab^
	9	7.61 ± 0.15 ^d^_d_	6.31 ± 0.10 ^c^_d_	5.81 ± 0.11 ^b^_d_	4.08 ± 0.08 ^a^_b_	4.12 ± 0.05 ^a^_b_
*E. coli*	0	1.78 ± 0.01 _a_	1.80 ± 0.02 _a_	1.72 ± 0.01 _a_	1.81 ± 0.02 _a_	1.76 ± 0.01 _a_
	3	1.89 ± 0.02 _b_	1.84 ± 0.02 _a_	1.81 ± 0.07 _a_	1.88 ± 0.01 _b_	1.83 ± 0.06 _a_
	6	3.1 ± 0.11 ^c^_c_	2.7 ± 0.12 ^b^_b_	2.15 ± 0.09 ^a^_b_	2.05 ± 0.16 ^a^_b_	2.08 ± 0.14 ^a^_b_
	9	3.8 ± 0.18 ^d^_d_	3.2 ± 0.07 ^c^_c_	2.70 ± 0.13 ^b^_c_	2.35 ± 0.15 ^a^_c_	2.29 ± 0.09 ^a^_c_

**Table 3:** Microbial changes in minced meat contaminated with *Listeria monocytogenes* Lhica 4 in the five different batches elaborated during storage time. Results are expressed as log cfu/g. Different superscript letters in the same row indicate significant differences (*p* < 0.05). Different subscript letters in the same column and microbial group indicate significant differences (*p* < 0.05).

Microbial Group	Storage Day	Batch				
		CM	SM	NM	AM	CHM
Total viable count	0	5.37 ± 0.04 _a_	5.40 ± 0.01 _a_	5.38 ± 0.03 _a_	5.41 ± 0.03 _a_	5.42 ± 0.01 _a_
	3	7.26 ± 0.11 ^d^_b_	6.55 ± 0.03 ^c^_b_	6.12 ± 0.14 ^b^_b_	5.46 ± 0.09 ^a^_a_	5.48 ± 0.05 ^a^_a_
	6	7.72 ± 0.20 ^c^_c_	6.75 ± 0.10 ^b^_c_	6.70 ± 0.17 ^b^_c_	6.00 ± 0.10 ^a^_b_	6.08 ± 0.07 ^a^_b_
	9	8.62 ± 0.21 ^d^_d_	7.65 ± 0.12 ^c^_d_	7.14 ± 0.08 ^b^_d_	6.62 ± 0.12 ^a^_c_	6.56 ± 0.09 ^a^_c_
Enterobacteriaceae	0	2.91 ± 0.02 _a_	2.89 ± 0.04 _a_	2.88 ± 0.03 _a_	2.89 ± 0.02 _a_	2.92 ± 0.02 _a_
	3	3.72 ± 0.10 ^b^_b_	3.32 ± 0.09 ^a^_b_	3.25 ± 0.08 ^a^_b_	3.27 ± 0.11 ^a^_b_	3.22 ± 0.19 ^a^_b_
	6	5.41 ± 0.09 ^dc^	4.41 ± 0.14 ^c^_c_	3.71 ± 0.10 ^b^_c_	3.24 ± 0.12 ^a^_b_	3.35 ± 0.08 ^a^_b_
	9	6.49 ± 0.05 ^d^_d_	5.21 ± 0.22 ^c^_d_	4.73 ± 0.07 ^b^_d_	3.30 ± 0.09 ^a^_b_	3.28 ± 0.05 ^a^_b_
*E. coli*	0	1.59 ± 0.01 _a_	1.60 ± 0.02 _a_	1.62 ± 0.01 _a_	1.60 ± 0.01 _a_	1.58 ± 0.04 _a_
	3	1.80 ± 0.04 _b_	1.84 ± 0.02 _b_	1.79 ± 0.07 _b_	1.82 ± 0.01 _b_	1.81 ± 0.06 _b_
	6	2.88 ± 0.10 ^c^_c_	2.23 ± 0.81 ^b^_b_	1.90 ± 0.06 ^a^_c_	1.85 ± 0.17 ^a^_b_	1.91 ± 0.10 ^a^_b_
	9	3.81 ± 0.10 ^d^_d_	3.09 ± 0.04 ^c^_c_	2.59 ± 0.13 ^b^_d_	2.12 ± 0.15 ^a^_c_	2.11 ± 0.09 ^a^_c_

**Table 4:** pH changes in minced meat added with the preservatives tested during storage. Different letters in the same row indicate significant differences (*p* < 0.05).

	Storage Day	Batch				
		CM	SM	NM	AM	CHM
pH	0	5.64 ± 0.12 ^b^	5.69 ± 0.07 ^b^	5.73 ± 0.04 ^b^	5.07 ± 0.06 ^a^	5.03 ± 0.09 ^a^
	3	6.51 ± 0.05 ^c^	6.31 ± 0.03 ^b^	6.33 ± 0.04 ^b^	5.38 ± 0.06 ^a^	5.40 ± 0.05 ^a^
	6	6.99 ± 0.03 ^c^	6.65 ± 0.10 ^b^	6.70 ± 0.07 ^b^	5.55 ± 0.05 ^a^	5.58 ± 0.07 ^a^
	9	7.42 ± 0.08 ^c^	7.05 ± 0.12 ^b^	7.10 ± 0.05 ^b^	5.92 ± 0.06 ^a^	5.96 ± 0.09 ^a^
